# Impact of increasing the levels of insoluble fiber and on the method of diet formulation measures of energy and nutrient digestibility in growing pigs

**DOI:** 10.1093/jas/skaa130

**Published:** 2020-04-21

**Authors:** Jesus A Acosta, Hans H Stein, John F Patience

**Affiliations:** 1 Department of Animal Science, Iowa State University, Ames, IA; 2 Department of Animal Sciences, University of Illinois, Urbana, IL

**Keywords:** amino acids, ileal-cannulation, insoluble fiber, soluble fiber, starch, swine

## Abstract

The objective of this study was to determine the differences in response to distillers dried grains with solubles (**DDGS**) level under constant nutrient or floating nutrient concentrations. A total of 21 ileal-cannulated gilts (33.1 ± 0.4 kg body weight) were randomly allotted to one of seven dietary treatments in a 3-period incomplete Latin square design (*n* = 9). Treatments consisted of a 0% DDGS basal diet, plus diets containing 15%, 30%, or 45% DDGS. Diets were formulated using one of two different formulation methods: 1) constant nutrient (**CNU**) where nutrients were held equal to the basal diet or 2) constant ingredients (**CIN**) where DDGS were added at the expense of corn and all other ingredients remained constant, so nutrient levels were allowed to “float.” Chromic oxide was added to the diets at 0.5% as an indigestible marker. Increasing the level of DDGS decreased the apparent ileal digestibility (**AID**) of dry matter (**DM**), gross energy (**GE**), starch, dispensable amino acids (**AA**), and fiber components (*P* < 0.050). The decrease in the AID of Lys, Met, Thr, and Trp was more pronounced under CNU compared with the CIN formulation method (*P* < 0.050). The decrease in the AID of hemicellulose was less pronounced under CNU compared with the CIN formulation method (*P* = 0.045). There was a DDGS level × formulation method interaction for the AID of acid hydrolyzed ether extract (**AEE**; *P* = 0.015); for the CNU formulation method, increasing level of DDGS decreased the AID of AEE from 0% to 30% and remained similar from 30% to 45% DDGS, whereas the CIN had no effect on the AID of AEE. Increasing the level of DDGS decreased the apparent total tract digestibility (**ATTD**) of DM, GE, and fiber components (*P* < 0.050), except for acid detergent fiber, which was not affected. The decrease in the ATTD of insoluble dietary fiber and total dietary fiber was less pronounced under CNU compared with CIN (*P* < 0.050). The ATTD of AEE decreased for CNU compared with CIN (*P* < 0.010). In conclusion, increasing the insoluble fiber level in the form of DDGS decreased the digestibility of most dietary components, including DM, GE, starch, insoluble fiber, and AA. The CNU and CIN formulation methods are equivalent when evaluating the digestibilities of DM, GE, starch, crude protein, and AA (when they were not added in purified synthetic forms). Differences between CNU and CIN formulation methods were detected for the digestibility of insoluble fiber, fat, and essential AA (when added as crystalline AA).

## Introduction

High fiber ingredients such as corn distillers dried grains with solubles (**DDGS**) are added to the diet because of their cost, availability, and nutrient profile. Insoluble fiber comprises most of the fiber present in DDGS and is characterized as being poorly digested and fermented in the gastrointestinal tract of pigs (Bach Knudsen, 2001; Gutierrez et al., 2013). Furthermore, increasing the addition of insoluble fiber in swine diets results in a decrease in the digestibility of other dietary components, such as gross energy (**GE**), dry matter (**DM**), amino acids (**AA**), and minerals (Lenis et al., 1996; Gutierrez et al., 2016). Thus, although there is a reasonable understanding of what insoluble fiber does, there is still a need to know if these effects are influenced by the way diets employed in such studies are formulated.

Experiments evaluating high fiber ingredients might employ diets formulated by adding the ingredient of interest at the expense of corn, with most other ingredients being held constant. In other words, nutrients are allowed to float—they are not held constant across all diets. This formulation method may have a potential flaw because changes in nutrient levels may confound the response of the pigs to the dietary treatments, even in a digestibility experiment. On the other hand, formulating to constant energy and nutrient levels will result in changes in other ingredients in the diets, and this could confound the response of the pigs to the experimental treatments; inadvertent effects occurring as a result of changing ingredient levels could affect the outcomes. However, in commercial practice, diets are formulated to constant energy and nutrient levels. As a result of these two scenarios, researchers are often faced with a serious dilemma on how to formulate experimental diets. The design of experimental diets typically falls between these two formulation methods (Benz et al., 2010; Lee et al., 2012; Gutierrez et al., 2016). Consequently, it would be useful to determine if the response to dietary treatment—in this case, fiber level—is different under a constant ingredien (**CIN**) compared with a constant nutrient (**CNU**) formulation approach.

Therefore, the objectives of this study were 1) to determine the impact of increasing the DDGS level, and this insoluble fiber, on the digestibility of energy and nutrients in growing pig diets and 2) to determine if the impact of fiber levels differ when diets are formulated to CNU or to CIN composition. We hypothesized that increasing the fiber level would decrease the digestibility of energy and nutrients. Additionally, we hypothesized that the CNU formulation method would be a better platform for evaluating fiber levels compared with the CIN method. Fiber levels were increased by increasing DDGS content in the diets, an approach that was deemed most logical due to their widespread use in American pig diets.

## Materials and Methods

All experimental procedures adhered to guidelines for the ethical and humane use of animals for research according to the Guide for the Care and Use of Agricultural Animals in Research and Teaching (FASS, 2010) and were approved by the Institutional Animal Care and Use Committee at Iowa State University (8-17-8584-S).

### Animals, housing, and experimental design

Twenty-one crossbred growing gilts (progeny of Camborough sows × 337 sires; PIC Inc., Hendersonville, TN) were surgically fitted with a T-cannula at the distal ileum following the procedures similar to those described by Stein et al. (1998). After recovery from surgery, pigs were weighed (33.1 ± 0.4 kg initial body weight) and randomly allotted to one of seven dietary treatments in a 3-period incomplete Latin square design, resulting in nine observations per treatment. Animals were housed in individual pens (1.2 × 1.5 m) in an environmentally controlled facility with a 12:12 (L:D) h cycle. Each pen was equipped with a feeder, a nipple waterer, and a half slated concrete floor. Treatments consisted of a basal diet without DDGS and diets containing 15%, 30%, or 45% DDGS (6.6% acid hydrolyzed ether extract [**AEE**]) as a source of insoluble fiber. Diets were formulated using one of the two methods (Table 1): CNU where the nutrient levels were maintained equal to those of the basal diet or CIN where DDGS were added at the expense of corn and all other ingredients remained constant; therefore, nutrients were then allowed to float. AA, vitamins, and minerals were added to all diets (Table 2) to meet or exceed the estimated requirement (NRC, 2012); levels of Thr were elevated as the inclusion of DDGS increased to account for a higher requirement in higher fiber diets (de Lange et al., 1989). Chromic oxide was added at 0.5% as an indigestible marker. All pigs were provided with the same daily amount of feed equivalent to 3.2 times the estimated requirement for maintenance energy (i.e., 197 kcal ME/kg0.6; NRC, 2012) of the average ME of the CNU and CIN; 30% DDGS diets. The daily feed allotments were divided into two equal meals that were provided at 0730 and 1630 hours. At the end of each collection period, all pigs were weighed, and daily feed allowance for the next collection period was adjusted. All diets were provided in mash form with ad libitum access to water.

**Table 1.** Ingredient composition of the experimental diets

		Formulation method					
	Basal	CNU^1^			CIN^2^		
	DDGS level, %						
Ingredients, %	0%	15%	30%	45%	15%	30%	45%
Corn	82.86	68.56	54.25	39.95	67.86	52.86	37.86
Corn DDGS-RO^3^	0.00	15.00	30.00	45.00	15.00	30.00	45.00
HP 300^4^	5.89	5.89	5.89	5.89	5.89	5.89	5.89
Casein	3.00	3.00	3.00	3.00	3.00	3.00	3.00
Plasma	3.00	3.00	3.00	3.00	3.00	3.00	3.00
Soybean oil	1.20	0.80	0.40	0.00	1.20	1.20	1.20
Limestone	1.29	1.37	1.45	1.53	1.29	1.29	1.29
Monocalcium phosphate	0.71	0.48	0.24	0.00	0.71	0.71	0.71
l-Lys HCl	0.39	0.32	0.25	0.18	0.39	0.39	0.39
dl-Met	0.06	0.04	0.02	0.00	0.06	0.06	0.06
Thr	0.11	0.07	0.04	0.00	0.11	0.11	0.11
Trp	0.03	0.02	0.01	0.00	0.03	0.03	0.03
Salt	0.60	0.60	0.60	0.60	0.60	0.60	0.60
Vitamin premix^5^	0.20	0.20	0.20	0.20	0.20	0.20	0.20
Trace mineral premix^6^	0.15	0.15	0.15	0.15	0.15	0.15	0.15
Chromic oxide	0.50	0.50	0.50	0.50	0.50	0.50	0.50

^1^CNU, DDGS were added at the expense of corn, and the nutrient levels were maintained equal to those of the basal diet.

^2^CIN, DDGS were added at the expense of corn, and all other ingredients were maintained equal to the basal diet; thus, nutrients were allowed to float.

^3^DDGS-RO, distillers dried grains with solubles-reduced oil.

^4^Processed soy protein concentrate (Hamlet Protein, Findlay, OH).

^5^Vitamin premix provided the following (per kg diet): 6,125 IU of vitamin A; 700 IU of vitamin D3; 50 IU of vitamin E; 3 mg of menadione (to provide vitamin K); 11 mg of riboflavin; 27 mg of d-pantothenic acid; 0.05mg of vitamin B12, and 56 mg of niacin.

^6^Mineral premix provided the following (per kg diet): 165 mg of Fe (ferrous sulfate); 165 mg of Zn (zinc sulfate); 39 mg of Mn (manganese sulfate); 16.5 mg of Cu (copper sulfate); 0.3 mg of I (calcium iodate); 0.3 mg of Se (sodium selenite); and 250 FTU/kg of phytase (Quantum Blue 5G, AB Vista Feed Ingredients; Marlborough, Wiltshire, UK).

**Table 2.** Analyzed chemical composition of the experimental diets (as-fed basis)

	Formulation method						
	Basal	CNU^1^			CIN^2^		
		DDGS level, %					
Item	0%	15%	30%	45%	15%	30%	45%
DM, %	87.3	87.7	88.0	88.4	87.8	87.6	88.4
GE, Mcal/kg	3.94	4.02	4.07	4.15	4.03	4.11	4.19
AEE, %	4.7	4.7	5.0	4.9	5.5	5.6	6.1
Starch, %	49.6	43.0	34.0	24.1	39.7	31.1	26.3
NDF, %	7.9	10.7	12.6	15.9	10.6	12.4	15.3
ADF, %	1.8	2.7	3.3	4.6	2.6	3.4	4.4
Insoluble hemicellulose, %	6.1	8.1	9.4	11.3	8.0	9.0	10.9
IDF, %	9.2	12.5	14.5	17.6	12.1	13.9	16.6
SDF, %	0.9	1.3	1.5	2.0	1.4	1.7	1.9
TDF^3^, %	10.1	13.8	16.2	19.6	13.5	15.6	18.6
Crude protein, %	14.8	18.3	21.6	25.1	18.8	21.7	25.4
Indispensable AA, %							
Arg	0.67	0.79	0.95	1.06	0.80	0.95	1.01
His	0.35	0.41	0.51	0.59	0.42	0.51	0.56
Ile	0.46	0.63	0.82	0.86	0.68	0.80	0.82
Leu	1.24	1.59	2.05	2.26	1.66	2.04	2.18
Lys	0.99	0.99	1.08	1.13	1.09	1.18	1.20
Met	0.28	0.31	0.35	0.40	0.33	0.37	0.41
Phe	0.66	0.72	0.98	1.12	0.81	0.97	1.07
Thr	0.64	0.71	0.79	0.89	0.73	0.84	0.94
Trp	0.20	0.20	0.22	0.24	0.21	0.23	0.26
Val	0.69	0.83	1.00	1.12	0.84	1.00	1.07
Sum of dispensable AA, %	6.83	8.16	10.05	11.63	8.35	10.06	11.23
Sum of all AA, %	13.00	15.36	18.80	21.30	15.90	18.94	20.75

^1^CNU, DDGS were added at the expense of corn, and the nutrient levels were maintained equal to those of the basal diet.

^2^CIN, DDGS were added at the expense of corn, and all other ingredients were maintained equal to the basal diet; thus, nutrients were allowed to float.

^3^TDF = SDF + IDF.

Pigs were reassigned to dietary treatment at the end of each collection period, but no pigs received a diet more than once. Each collection period involved 9 d of adaptation to dietary treatments followed by 2 d of feces subsample collection and 3 d of ileal digesta subsample collection (Gutierrez et al., 2016).

### Sample collection, chemical analyses, and calculations

Ten diet subsamples were randomly collected at the feed mill at the time of mixing and then thoroughly homogenized and carefully subsampled. Fresh fecal subsamples were obtained from individual pigs via grab sampling. Ileal subsamples were collected by attaching a 207-mL plastic bag (Whirl-Pak; Nasco, Fort Atkinson, WI) to the opened cannula with a cable tie. Bags were removed once they were filled with digesta or at least every 30 min for 8 h per collection day. All subsamples were stored at –20 °C to avoid bacterial or chemical degradation.

Prior to being assayed, fecal subsamples were thawed and oven-dried in a convection oven at 65 °C until subsamples reached constant weight (Jacobs et al., 2011); ileal subsamples were lyophilized. Diets and dried ileal and fecal subsamples were ground in a Wiley Mill (Variable Speed Digital ED-5 Wiley Mill; Thomas Scientific, Swedesboro, NJ) through a 1-mm screen and stored in desiccators to maintain a constant percentage of DM.

Chemical analysis of diets, feces, and ileal digesta was performed at the Monogastric and Comparative Nutrition Laboratory (Iowa State University, Ames, IA), the Monogastric Nutrition Laboratory (University of Illinois-Champaign, IL) and the Ajinomoto Heartland, Inc., Amino Acid Laboratory (Eddyville, IA). DM was determined using a drying oven (method 930.15; AOAC, 2007). AEE was assayed using a SoxCap hydrolyzer (model SC 247) and a Soxtec fat extractor (model 255; Foss, Eden Prairie, MN; method 968; AOAC, 2007). Starch (diets and ileal samples only) was analyzed using a Megazyme total starch assay kit (Wicklow, Ireland; modified method 996.11; AOAC, 1996). Insoluble dietary fiber (**IDF**) and soluble dietary fiber (**SDF**) were determined using the Ankom TDF Dietary Fiber Analyzer (AOAC 991.43; AOAC, 2007; Ankom Technology, Macedon, NY). Total dietary fiber (**TDF**) was reported as the sum of IDF and SDF. Acid detergent fiber (**ADF)** and neutral detergent fiber (**NDF)** were determined using an Ankom automated fiber analyzer (model 2000, Macedon, NY; a modified method from Van Soest and Robertson, 1979). Insoluble hemicellulose concentration was determined by subtracting ADF from NDF. GE was determined using a bomb calorimeter (model 6200; Parr Instrument Co., Moline, IL). Benzoic acid (6,318 kcal/kg; Parr Instruments, Moline, IL) was used as the standard for calibration and was determined to contain 6,319 ± 2.2 kcal/kg. Chromium was determined using the method of Fenton and Fenton (1979); absorption was measured at 440 nm using a spectrophotometer (Synergy 4; BioTek Instruments Inc., Winooski, VT). Crude protein as N × 6.25 was determined by using an N analyzer (Rapid N Cube, Elementar Analysensysteme GmbH, Hanau, Germany; method 990.03, AOAC, 2006). AA were analyzed according to method 982.30 E (a, b, c; AOAC, 2007) using an Amino Acid Analyzer (model L 8800; Hitachi High Technologies America Inc., Pleasanton, CA).

The apparent ileal digestibility (**AID**) of starch, crude protein, AA, DM, GE, AEE, IDF, SDF, TDF, NDF, ADF and hemicellulose and the apparent total tract digestibility (**ATTD**) of DM, GE, AEE, IDF, SDF, TDF, NDF, ADF, and hemicellulose were determined using the following equation (Oresanya et al., 2008):

$$\begin{array}{c} \text{ATTD}\;\text{or}\;\text{AID},\;\%\;=\;[\text{1}00-[\text{1}00\;\times \;(\%\;\text{chromic}\;\text{oxide}\;\text{in}\;\text{feed}\;/\;\%\;\text{chromic}\;\text{oxide}\;\text{in}\\ \text{feces}\;\text{or}\;\text{ileal}\;\text{digesta})\;\times \;(\text{concentration}\;\text{of}\;\text{component}\;\text{in}\;\text{feces}\;\text{or}\;\text{ileal}\;\text{digesta}\;/\;\\ \text{concentration}\;\text{of}\;\text{component}\;\text{in}\;\text{feed})]] \end{array}$$

Hindgut disappearance of DM, GE, AEE, IDF, SDF, TDF, NDF, ADF, and hemicellulose was calculated as the difference between the concentration of these components in the ileal digesta and the feces (Pilcher et al., 2013).

### Statistical analysis

Data were analyzed according to the following model:

$$y_{ijkl}=\;\mu +\tau_{i}+\lambda_{j}+{\left(\tau \lambda \right)}_{ij}+\delta_{k}+\;\theta_{l}+\epsilon_{ijkl}$$

where y_ijkl_ represents the observed value, μ is the overall mean, τ represents the fixed effect of DDGS level, λ represents the fixed effect of formulation method, τλ represents the interaction between DDGS level and formulation method, δ represents the random effect of pig (0 ~Ν (0,σ2δ ), θ represents the random effect of the collection period [0 ~Ν (0, σ2θ)], and ϵ is the random error [0 ~Ν (0,σ2ε)].

The UNIVARIATE procedure of SAS (SAS Inst., Inc., Cary, NC) was used to analyze for normality and outliers (defined as standardized residuals greater than three standard deviations away from the mean). The model was analyzed using the MIXED procedure of SAS. For significant interactions, the Fisher’s Least Significant Difference adjustment was used to assess pairwise comparisons between individual treatment means. Additionally, the CONTRAST statement was used to compare the average response to DDGS levels in CNU and CIN against the basal formulation. Effects were considered statistically significant with *P*-values ≤ 0.050, and *P*-values between 0.050 and 0.100 were considered trends. The pig was the experimental unit for all analyses.

## Results

All animals were successfully cannulated in the distal ileum and recovered from surgery without incident. All pigs fully consumed their daily rations during the entire experimental period.

Except for the average of dispensable AA, the AID of crude protein, the average of all AA, the average of all indispensable AA, and the AID of each indispensable amino acid decreased as the level of DDGS increased (*P* < 0.010; Table 3). The formulation method did not affect the AID of crude protein, Arg, His, Ile, Leu, Phe, Val, the average of dispensable AA, and the average of all AA. However, the CNU formulation method decreased the AID of Lys and Met compared with the CIN formulation method (*P* = 0.001 and *P* = 0.013, respectively). There was a DDGS level × formulation method interaction for the AID of Thr and Trp (*P* = 0.050 and P = 0.029, respectively); the decrease in AID from 0% to 30% DDGS was not different between the CNU and the CIN formulation methods. However, the AIDs of Thr and Trp decreased further for the CNU 45% DDGS diet (*P* < 0.050), but it did not decrease further to the CIN 45% DDGS diet.

**Table 3.** Impact of DDGS level and formulation method on the AID of crude protein and AA

		Formulation method										Contrast^3^	
	Basal	CNU^1^			CIN^2^				*P*-value				
	DDGS level, %												
Item	0	15	30	45	15	30	45	SEM	Level	Formula	Level × Formula	Basal vs. CNU	Basal vs. CIN
AID, %													
Crude protein	76.9	74.8	73.4	71.5	74.8	73.2	73.2	0.8	0.002	0.329	0.230	<0.001	<0.001
Indispensable AA	82.5	79.3	78.3	75.5	79.4	78.9	76.9	0.7	<0.001	0.120	0.479	<0.001	<0.001
Arg	85.5	84.0	83.8	82.4	83.4	83.9	82.8	0.5	0.030	0.969	0.547	0.001	0.002
His	83.7	80.2	79.5	76.8	79.7	79.4	76.6	1.0	<0.001	0.550	0.938	<0.001	<0.001
Ile	77.0	77.7	78.0	73.8	79.0	78.1	74.4	0.8	<0.001	0.185	0.648	0.530	0.844
Leu	83.9	82.9	83.7	81.7	83.4	83.8	82.0	0.7	0.001	0.415	0.957	0.074	0.248
Lys	86.6	81.4	79.2	74.7	83.0	81.2	77.0	0.7	<0.001	0.001	0.855	<0.001	<0.001
Met	88.6	86.2	84.5	82.9	86.2	85.6	84.6	0.5	<0.001	0.013	0.169	<0.001	<0.001
Phe	83.6	79.4	81.7	80.1	81.0	81.7	80.4	0.7	0.007	0.122	0.273	<0.001	0.002
Thr	79.5^a^	75.0^b^	72.0^c^	68.8^d^	74.5^b^	73.3^bc^	71.9^c^	0.8	<0.001	0.022	0.050	<0.001	<0.001
Trp	76.6^a^	69.9^b^	68.0^c^	63.4^d^	70.3^b^	68.5^bc^	67.9^c^	0.9	<0.001	0.004	0.029	<0.001	<0.001
Val	80.0	77.0	77.3	73.9	77.0	77.0	74.2	0.8	<0.001	0.920	0.645	<0.001	<0.001
Dispensable AA^4^	77.1	73.8	74.8	72.5	73.9	74.6	73.2	1.0	0.133	0.514	0.551	<0.001	0.001
All AA^5^	80.6	77.6	78.1	75.3	77.9	78.1	76.1	0.8	0.002	0.345	0.568	<0.001	<0.001

^1^CNU, DDGS were added at the expense of corn, and the nutrient levels were maintained equal to those of the basal diet.

^2^CIN, DDGS were added at the expense of corn, and all other ingredients were maintained equal to the basal diet; thus, nutrients were allowed to float.

^3^Basal diet vs. CINdiets and basal diet vs. CNU diets.

^4^Average AID for all dispensable AA.

^5^Average AID for all AA (indispensable and dispensable).

^a–d^Means within a row with different superscripts significantly differ (*P* < 0.050).

Averaged across the DDGS levels, the AID of crude protein, indispensable AA, Arg, His, Lys, Met, Phe, Thr, Trp, Val, dispensable AA, and the average of all AA decreased using either CNU or CIN compared with the basal diet (*P* < 0.010). However, the AID of Ile was not different for the average response to DDGS using either CNU or CIN. The average AID of Leu tended to be reduced as DDGS increased for the CNU, but in the CIN formulation method, no difference was observed.

The AID of DM, GE, IDF, SDF, TDF, NDF, hemicellulose, and starch decreased as the level of DDGS increased (*P* < 0.050; Table 4). In contrast, the AID of ADF increased as the level of DDGS increased (*P* = 0.027). The formulation method did not affect the AID of DM, GE, IDF, TDF, ADF, and starch. However, using the CNU formulation method resulted in a tendency for increased AID of IDF and increased the AID of hemicellulose compared with the CIN method (*P* = 0.090 and *P* = 0.045, respectively). For the AID of AEE, there was a DDGS level × formulation method interaction (*P* = 0.015); with the CNU formulation method, the AID of AEE decreased from 0% to 30 % DDGS and remained constant from 30% to 45% DDGS. In contrast, for the CIN formulation method, the AID of AEE did not change from 0% to 45% DDGS.

**Table 4.** Impact of DDGS level and formulation method on the AID, the ATTD, and hindgut disappearance of dietary components

	Formulation method											Contrast^3^	
	Basal	CNU^1^			CIN^2^				*P*-value				
	DDGS level, %												
Item	0	15	30	45	15	30	45	SEM	Level	Formula	Level × Formula	Basal vs. CNU	Basal vs. CIN
AID, %													
DM	79.1	72.4	66.0	61.5	72.0	66.2	61.5	0.8	<0.001	0.225	0.160	<0.001	<0.001
GE	80.4	74.3	69.3	64.1	74.2	69.6	66.5	0.8	<0.001	0.102	0.113	<0.001	<0.001
AEE	73.9^a^	70.9^b^	67.8^c^	68.1^c^	73.6^a^	74.6^a^	75.4^a^	0.9	0.472	<0.001	0.015	<0.001	<0.189
IDF	42.5	33.2	24.7	21.9	32.3	20.1	21.0	2.4	<0.001	0.204	0.579	<0.001	<0.001
SDF	32.0	27.5	18.2	8.9	38.9	16.9	18.6	4.5	<0.001	0.073	0.322	0.016	0.014
TDF^4^	41.5	32.5	23.5	20.6	33.0	19.3	20.9	2.1	<0.001	0.473	0.412	<0.001	<0.001
NDF	40.3	34.0	24.9	22.4	31.3	21.3	20.1	2.4	<0.001	0.090	0.818	<0.001	<0.001
ADF	5.2	3.9	2.1	9.6	3.2	3.0	7.3	3.1	0.027	0.709	0.778	0.991	0.710
Insoluble hemicellulose	50.6	43.6	32.8	27.5	40.3	28.1	26.6	2.3	<0.001	0.045	0.556	<0.001	<0.001
Starch	95.8	94.9	92.8	93.1	94.0	93.1	93.2	0.8	0.014	0.684	0.504	0.002	<0.001
ATTD, %													
DM	85.7	82.7	78.7	73.7	82.4	78.0	74.3	0.5	<0.001	0.732	0.326	<0.001	<0.001
GE	84.8	82.3	78.6	74.4	82.2	78.5	75.6	0.5	<0.001	0.337	0.226	<0.001	<0.001
AEE	61.9	61.8	60.7	60.1	65.4	66.2	67.5	1.2	0.931	<0.001	0.170	0.354	0.017
IDF	53.7	54.4	46.1	41.3	50.3	41.8	40.2	1.3	<0.001	0.004	0.340	<0.001	<0.001
SDF	73.7	82.6	83.3	87.8	80.0	82.7	82.2	2.8	0.405	0.201	0.662	0.002	0.007
TDF	55.5	57.1	50.0	45.9	53.3	46.3	44.5	1.2	<0.001	0.004	0.493	0.002	<0.001
NDF	53.3	50.4	44.0	39.3	49.9	41.0	39.9	1.3	<0.001	0.324	0.328	<0.001	<0.001
ADF	29.8	32.8	30.1	34.4	32.3	32.3	33.5	1.9	0.283	0.879	0.611	0.190	0.294
Insoluble hemicellulose	60.1	55.8	47.7	41.5	54.2	44.1	42.4	1.2	<0.001	0.114	0.171	<0.001	<0.001
Hindgut disappearance, %													
DM	6.6	10.5	12.6	14.3	10.4	11.8	12.8	1.0	0.003	0.217	0.672	<0.001	<0.001
GE	4.4	8.0	9.3	10.3	7.9	8.9	9.1	1.0	0.091	0.383	0.722	<0.001	<0.001
AEE	−9.5	−8.4	−6.9	−7.8	−7.8	−8.5	−7.7	2.1	0.949	0.947	0.867	0.417	0.382
IDF	10.6	21.1	21.5	19.4	18.0	21.5	19.4	2.9	0.604	0.582	0.738	0.002	0.002
SDF	42.2	54.9	64.4	78.8	40.7	65.7	63.9	4.5	<0.001	0.019	0.166	<0.001	<0.001
TDF	13.5	24.4	26.6	25.4	20.4	26.7	24.0	2.5	0.197	0.320	0.652	<0.001	<0.001
NDF	12.5	16.2	19.9	16.9	18.3	19.6	19.2	2.9	0.541	0.438	0.828	0.074	0.018
ADF	23.9	28.7	28.3	25.1	29.1	29.3	26.1	3.9	0.431	0.715	0.993	0.362	0.295
Insoluble hemicellulose	9.0	12.2	15.0	14.1	13.8	16.0	15.8	2.6	0.460	0.380	0.977	0.075	0.017

^1^CNU, DDGS were added at the expense of corn, and the nutrient levels were maintained equal to those of the basal diet.

^2^CIN, DDGS were added at the expense of corn, and all other ingredients were maintained equal to the basal diet; thus, nutrients were allowed to float.

^3^Basal diet vs. CINdiets and basal diet vs. CNU diets.

^4^TDF = SDF + IDF.

^a–c^Means within a row with different superscripts significantly differ (*P* < 0.050).

The AID of DM, GE, IDF, SDF, TDF, NDF, and insoluble hemicellulose decreased for the average response to DDGS using both the CNU and the CIN formulation methods compared with the basal diet (*P* < 0.050). The AID of AEE decreased for the average response to DDGS if diets were formulated based on CNU (*P* < 0.001) but did not change when the CIN method was used. The AID of ADF was not different between the CNU and CIN formulation methods compared with the basal diet.

The ATTD of DM, GE, IDF, TDF, NDF, and insoluble hemicellulose decreased as the level of DDGS increased in the formulation (*P* < 0.001). In contrast, the level of DDGS did not affect the ATTD of AEE, SDF, and ADF. Formulating using the CNU method resulted in reduced ATTD of AEE compared with the CIN method. In contrast, formulating diets using the CNU method resulted in an increase in the ATTD of IDF and TDF compared with results obtained using the CIN method (*P* = 0.004 and *P* = 0.004, respectively). The ATTD of DM, GE, IDF, SDF, TDF, NDF, and hemicellulose decreased for the average response to DDGS using the CNU and the CIN formulation methods compared with the basal diet (*P* < 0.050). The ATTD of AEE was similar for the average response to DDGS using the CNU method but increased using the CIN method compared with the basal diet (*P* = 0.017). The ATTD of ADF was not different between the CNU and CIN formulation methods.

The hindgut disappearance of DM increased, whereas the hindgut disappearance of GE tended to increase as the level of DDGS increased in the formulation (*P* = 0.003 and *P* = 0.091, respectively). Likewise, the hindgut disappearance of SDF increased as the level of DDGS increased (*P* < 0.001). The hindgut disappearance of IDF, TDF, NDF, ADF, and hemicellulose was not affected by the DDGS level. Using the CNU formulation method increased the hindgut disappearance of SDF compared with the CIN formulation method. However, the formulation method did not affect the hindgut disappearance of DM, GE, IDF, TDF, NDF, ADF, and hemicellulose. The hindgut disappearance of DM, GE, IDF, SDF, and TDF increased for the average response to DDGS level compared with the basal diet (*P* < 0.010). Similarly, the hindgut disappearance of NDF and hemicellulose tended to increase for CNU compared with the basal diet (*P* = 0.074 and *P* = 0.075, respectively) and increased for CIN compared with the basal diet (*P* = 0.018 and *P* = 0.017, respectively).

## Discussion

As with any ingredient, corn DDGS are added to diets when they are available at a competitive cost. When formulated correctly, performance using high levels of DDGS can be maintained throughout the grow-finish period (Weber et al., 2015). Although variable in chemical composition (Stein and Shurson, 2009), DDGS are not only considered a good source of GE, total AA, fat, phosphorus but also have a high insoluble fiber concentration (Patience and Petry, 2019). However, the efficiency in the utilization of most of these dietary components is moderate, reducing its potential feeding value (Stein and Shurson, 2009). The decreased digestibilities of DM and GE when DDGS is added to swine diets have been attributed to the increased concentration of insoluble fiber (Gutierrez et al., 2013). In fact, in this as well as similar experiments (Gutierrez et al., 2016), a major proportion of the decrease in digestibility can be attributed to the poor use of insoluble fiber by growing pigs; this is reflected in the amount of DM excreted in the feces.

Increasing the DDGS level decreased the AID of SDF in the small intestine but increased the hindgut disappearance of SDF in the large intestine. These results agree with Jaworski and Stein (2017), who compared a basal corn–soybean meal diet and a diet with 30% DDGS. These data indicate that although soluble fiber represents only a minor component in DDGS, it is extensively used in the large intestine regardless of the DDGS level.

The other proportion of the decrease in the digestibility of DM and GE is the result of the decrease in the digestibility of other dietary components such as starch and AA. The high digestibility of starch in pigs is mainly attributed to effective enzymatic digestive mechanisms along the small intestine (Li et al., 2015). Results of this experiment confirm that although starch is well digested in the small intestine (about 95%), the increased level of insoluble fiber modestly decreased starch digestibility. Starch digestibility can be affected by insoluble fiber level (Rosenfelder-Kuon et al., 2017), mainly attributed to encapsulation within the cell wall components, which remain in DDGS following the fermentation. It is expected that starch in DDGS is less digestible than that found in corn since starch in DDGS is the residual fraction resistant to fermentation in the distillery process; this, in turn, probably means it would be resistant to digestion in the small intestine of the pig as well.

The observation that increasing the level of DDGS decreased the AID of AA (except for the average of dispensable AA) may be attributed to insoluble fiber “trapping” nutrients and limiting their exposure to digestive enzymes, especially those directly associated with cell walls or encapsulated in the cell wall matrix (Kerr and Shurson, 2013; Grundy et al., 2016); this, in turn, decreases estimates of nutrient digestibility (Liu et al., 2014). In fact, data from Li et al. (1994) support the fiber trapping mechanism to explain the decrease in the digestibility of AA because there was no effect on the AID of AA by adding a crystalline source of insoluble fiber to the diet.

Ingredients high in IDF, including corn DDGS, are bulkier than corn or soybean meal (Wu et al., 2016) and thus increase intestinal swelling and mucus secretions (Bach Knudsen et al., 2012) and also dilute nutrients embedded in the digesta. Insoluble fiber may also increase the intestinal rate of passage (Wenk, 2001; Lindberg, 2014), which reduces the time of exposure to the digestive processes. However, it is also possible that fiber-independent factors such as heat-damaged AA in DDGS can also contribute to the decrease in the use of AA (Columbus and de Lange, 2012).

The other factor studied in this experiment was the formulation method. In practical terms, the difference between the CNU and CIN formulation methods was a slight change in the chemical profile of the diet: more ether extract, more indispensable AA, and more macro minerals in diets formulated based on CIN compared with CNU. However, in all instances, the level of these nutrients achieved or exceeded the pigs’ requirement (NRC, 2012). Independent of the DDGS level, digestibility differences between both formulation methods were associated with three diet fractions: insoluble fiber, some indispensable AA, and the AEE. Changes in the digestibility of insoluble fiber are a direct consequence of the microbiota’s ability to ferment cell wall components (Sciellour et al., 2018), a consequence of the microbiota shifting and adjusting according to the substrate present in their environment (Hillman et al., 2017; Williams et al., 2017). Any observed differences between the two formulation methods can be attributed to the changes in diet composition that may shift the profile of the nutrients reaching the terminal ileum and the fermentation of the insoluble fiber fractions. This experiment suggests that CIN diets (more AA, fat, and minerals than CNU diets) decrease the digestibility of insoluble fiber. Considering that the chemical composition profile of the CIN diets is unlikely to be formulated under practical conditions, and there is no practical way to adjust their effect on fiber digestibility, the CIN formulation method is not recommended to evaluate fiber utilization. Thus, the CNU formulation method is recommended to determine the fiber digestibility of an ingredient.

Although the AID of Lys, Met, Thr, and Trp decreased as the level of DDGS increased, the reduction was more pronounced in the CNU than the CIN formulation method. This difference can be attributed to the addition of synthetic AA. In the CNU formulation method, the level of synthetic AA decreased, whereas, in the CIN formulation method, the level of synthetic AA was kept constant across DDGS levels. Unlike dietary AA present in ingredients in the form of proteins, crystalline AA are readily available and highly digestible because they are not trapped in the ingredient fiber matrix. Therefore, the use of the CIN formulation method is recommended when measuring AA digestibility, as it eliminates the confounding effect of synthetic AA when evaluating the response to DDGS.

Increasing the level of DDGS using the CNU formulation method (maintaining similar AEE level across diets) decreased the AID of AEE, whereas when DDGS level increased using the CIN method (increased level of AEE as a result of the constant addition of soybean oil across diets) resulted in similar AID of AEE. On the other hand, increasing the level of DDGS did not influence the ATTD of AEE, but was lower using the CNU compared with the CIN formulation method. The apparent digestibility of AEE can be confounded by both nutrients AND ingredients. Some research suggests that extracted fat is more digestible than the fat present in ingredients (Kil et al., 2010, 2011), supporting the need for constant addition of soybean oil (using the CIN formulation method to avoid the confounding effect of ingredient). However, this differential in the digestibility estimates may be an artifact of the experimental design. The intestinal endogenous losses can substantially affect the apparent digestibility of AEE more at lower levels of inclusion than at higher levels (Jørgensen et al., 1993). In fact, Gutierrez et al. (2016) demonstrated that soybean oil did not affect the true digestibility of fat. A solution to compare apparent digestibility is to have similar fat levels across diets (achieved using the CNU formulation method); otherwise, digestibility values need to be corrected for endogenous losses to avoid biased comparisons.

In conclusion, increasing IDF in diets for pigs by adding DDGS decreased the digestibility of most dietary components, including DM, GE, starch, IDF, and AA. The CNU and CIN formulation methods are equivalent when evaluating the digestibility of DM, GE, starch, crude protein, and AA (when they were not added in purified-synthetic forms). However, differences between CNU and CIN formulation methods were detected for the digestibility of insoluble fiber, fat, and essential AA (when added in a purified-synthetic form). On the basis of these results, the CNU formulation method is suggested for use when evaluating the digestibility of insoluble fiber and fat. In contrast, the CIN method is recommended for use when evaluating AA digestibility of an ingredient if synthetic AA are added in the formulation.

## Abbreviations

AA: amino acids
ADF: acid detergent fiber
AEE: acid hydrolyzed ether extract
AID: apparent total tract digestibility
ATTD: apparent ileal digestibility
CIN: constant ingredients
CNU: constant nutrients
DDGS: distillers dried grains with solubles
DM: dry matter
GE: gross energy
IDF: insoluble dietary fiber
NDF: neutral detergent fiber
SDF: soluble dietary fiber
TDF: total dietary fiber

## References

[CIT0001] AOAC 1996 Official methods of analysis. Washington (DC): Association of Official Analytical Chemists.

[CIT0002] AOAC 2006 Official methods of analysis of AOAC International. 17th ed. Gaithersburg (MD): AOAC International.

[CIT0003] AOAC 2007 Official methods of analysis of AOAC International. 18th ed. Gaithersburg (MD): AOAC International.

[CIT0004] Bach Knudsen K. E 2001 The nutritional significance of ‘dietary fibre’ analysis. Anim. Feed Sci. Tech. 90:3–20. doi:10.1016/S0377-8401(01)00193-6

[CIT0005] Bach Knudsen K. E., Hedemann M. S., and Laerke H. N.. 2012 The role of carbohydrates in intestinal health of pigs. Anim. Feed Sci. Technol. 173:41–53. doi:10.1016/j.anifeedsci.2011.12.020

[CIT0006] Benz J. M., Linneen S. K., Tokach M. D., Dritz S. S., Nelssen J. L., DeRouchey J. M., Goodband R. D., Sulabo R. C., and Prusa K. J.. 2010 Effects of dried distillers grains with solubles on carcass fat quality of finishing pigs. J. Anim. Sci. 88:3666–3682. doi:10.2527/jas.2010-2937 20656980

[CIT0007] Columbus D., and de Lange C. F.. 2012 Evidence for validity of ileal digestibility coefficients in monogastrics. Br. J. Nutr. 108(Suppl 2):S264–S272. doi:10.1017/S0007114512002334 FASS. 2010 Guide for the care and use of agricultural animals in research and teaching. 3rd ed Champaign (IL): Federation of Animal Science Societies; p. 169. 23107537

[CIT0008] Fenton T. W., and Fenton M.. 1979 An improved procedure for the determination of chromic oxide in feed and feces. Can. J. Anim. Sci. 59:631–634. doi:10.4141/cjas79-081

[CIT0009] Grundy M. M., Edwards C. H., Mackie A. R., Gidley M. J., Butterworth P. J., and Ellis P. R.. 2016 Re-evaluation of the mechanisms of dietary fibre and implications for macronutrient bioaccessibility, digestion and postprandial metabolism. Br. J. Nutr. 116:816–833. doi:10.1017/S0007114516002610 27385119 PMC4983777

[CIT0010] Gutierrez N. A., Kerr B. J., and Patience J. F.. 2013 Effect of insoluble-low fermentable fiber from corn-ethanol distillation origin on energy, fiber, and amino acid digestibility, hindgut degradability of fiber, and growth performance of pigs. J. Anim. Sci. 91:5314–5325. doi:10.2527/jas.2013-6328 24045479

[CIT0011] Gutierrez N. A., Serão N. V., and Patience J. F.. 2016 Effects of distillers’ dried grains with solubles and soybean oil on dietary lipid, fiber, and amino acid digestibility in corn-based diets fed to growing pigs. J. Anim. Sci. 94:1508–1519. doi:10.2527/jas.2015-9529 27136010

[CIT0012] Hillman E. T., Lu H., Yao T., and Nakatsu C. H.. 2017 Microbial ecology along the gastrointestinal tract. Microbes Environ. 32:300–313. doi:10.1264/jsme2.ME17017 29129876 PMC5745014

[CIT0013] Jacobs B. M., Patience J. F., Dozier W. A. 3rd, Stalder K. J., and Kerr B. J.. 2011 Effects of drying methods on nitrogen and energy concentrations in pig feces and urine, and poultry excreta. J. Anim. Sci. 89:2624–2630. doi:10.2527/jas.2010-3768 21454859

[CIT0014] Jaworski N. W., and Stein H. H.. 2017 Disappearance of nutrients and energy in the stomach and small intestine, cecum, and colon of pigs fed corn-soybean meal diets containing distillers dried grains with solubles, wheat middlings, or soybean hulls. J. Anim. Sci. 95:727–739. doi:10.2527/jas.2016.0752 28380581

[CIT0015] Jørgensen H., Jakobsen K., and Eggum B. O.. 1993 Determination of endogenous fat and fatty acids at the terminal ileum and on faeces in growing pigs. Acta Agric. Scand. A Anim. Sci. 43:101–106. doi:10.1080/09064709309410151

[CIT0017] Kerr B. J., and Shurson G. C.. 2013 Strategies to improve fiber utilization in swine. J. Anim. Sci. Biotechnol. 4:11. doi:10.1186/2049-1891-4-11 23497595 PMC3623846

[CIT0018] Kil D. Y., Ji F., Stewart L. L., Hinson R. B., Beaulieu A. D., Allee G. L., Patience J. F., Pettigrew J. E., and Stein H. H.. 2011 Net energy of soybean oil and choice white grease in diets fed to growing and finishing pigs. J. Anim. Sci. 89:448–459. doi:10.2527/jas.2010-3233 20971888

[CIT0019] Kil D. Y., Sauber T. E., Jones D. B., and Stein H. H.. 2010 Effect of the form of dietary fat and the concentration of dietary neutral detergent fiber on ileal and total tract endogenous losses and apparent and true digestibility of fat by growing pigs. J. Anim. Sci. 88:2959–2967. doi:10.2527/jas.2009-2216 20453088

[CIT0020] de Lange C. F., Sauer W. C., Mosenthin R., and Souffrant W. B.. 1989 The effect of feeding different protein-free diets on the recovery and amino acid composition of endogenous protein collected from the distal ileum and feces in pigs. J. Anim. Sci. 67:746–754. doi:10.2527/jas1989.673746x 2722704

[CIT0021] Lee J. W., McKeith F. K., and Stein H. H.. 2012 Up to 30% corn germ may be included in diets fed to growing-finishing pigs without affecting pig growth performance, carcass composition, or pork fat quality. J. Anim. Sci. 90:4933–4942. doi:10.2527/jas.2012-5129 22851251

[CIT0022] Lenis N. P., P. Bikker, J. van der Meulen, J. Th. M. van Diepen, J. G. M. Bakker, and A. W. Jongbloed. 1996. Effect of neutral detergent fiber on ileal digestibility and portal flux of nitrogen and amino acids and on nitrogen utilization in growing pigs. J. Anim. Sci. 74:2687–2699. doi:10.2527/2001.7992418x 8923183

[CIT0023] Li S., Sauer W. C., and Hardin R. T.. 1994 Effect of dietary fibre level on amino acid digestibility in young pigs. Can. J. Anim. Sci. 74:327–333. doi:10.4141/cjas94-044 7928753

[CIT0024] Li Y., Zhang A. R., Luo H. F., Wei H., Zhou Z., Peng J., and Ru Y. J.. 2015 In vitro and in vivo digestibility of corn starch for weaned pigs: effects of amylose:amylopectin ratio, extrusion, storage duration, and enzyme supplementation. J. Anim. Sci. 93:3512–3520. doi:10.2527/jas.2014-8790 26440020

[CIT0025] Lindberg J. E 2014 Fiber effects in nutrition and gut health in pigs. J. Anim. Sci. Biotechnol. 5:15. doi:10.1186/2049-1891-5-15 24580966 PMC3975931

[CIT0026] Liu Y., Song M., Almeida F. N., Tilton S. L., Cecava M. J., and Stein H. H.. 2014 Energy concentration and amino acid digestibility in corn and corn coproducts from the wet-milling industry fed to growing pigs. J. Anim. Sci. 92:4557–4565. doi:10.2527/jas.2014-6747 25267997

[CIT0029] NRC 2012 Nutrient requirements of swine. 11th rev. ed. Washington (DC): The National Academies Press.

[CIT0030] Oresanya T. F., Beaulieu A. D., and Patience J. F.. 2008 Investigations of energy metabolism in weanling barrows: the interaction of dietary energy concentration and daily feed (energy) intake. J. Anim. Sci. 86:348–363. doi:10.2527/jas.2007-0009 17998419

[CIT0031] Patience J. F., and Petry A. L.. 2019 Susceptibility of fibre to exogenous carbohydrases and impact on performance in swine. In: Gonzalez-Ortiz G., Bedford M. R., Bach Knudsen K. E., Courtin C., and Classen H. L., editors. The value of fibre. Engaging the second brain for animal nutrition. Wageningen (The Netherlands): Wageningen Academic Press; p. 101–117.

[CIT0032] Pilcher C. M., Arentson R., and Patience J. F.. 2013 Impact of tylosin phosphate and distillers dried grains with solubles on energy and nutrient digestibility and flow through the gastrointestinal tract in growing pigs. J. Anim. Sci. 91:5687–5695. doi:10.2527/jas.2013-6746 24126274

[CIT0033] Rosenfelder-Kuon P., Strang E. J. P., Spindler H. K., Eklund M., and Mosenthin R.. 2017 Ileal starch digestibility of different cereal grains fed to growing pigs. J. Anim. Sci. 95:2711–2717. doi:10.2527/jas.2017.1450 28727064

[CIT0034] Sciellour M., Labussière E., Zemb O., Renaudeau D.. 2018 Effect of dietary fiber content on nutrient digestibility and fecal microbiota composition in growing-finishing pigs. PLoS One.13:1–20. doi:10.1371/journal.pone.0206159 PMC6200266 30356293

[CIT0035] Stein, H. H., and G. C. Shurson. 2009. Board-invited review: the use and application of distillers dried grains with solubles in swine diets. J. Anim. Sci. 87:1292–1303. doi:10.2527/jas.2008-0966 19028847

[CIT0036] Stein H. H., Shipley C. F., and Easter R. A.. 1998 Technical note: a technique for inserting a T-cannula into the distal ileum of pregnant sows. J. Anim. Sci. 76:1433–1436. doi:10.2527/1998.7651433x 9621950

[CIT0037] Van Soest, P. J., and J. B. Robertson. 1979. Systems of analysis for evaluating fibrous feeds. In: W. J. Pigden, C. C. Balch, and Michael Graham, editors. Proceedings of the International Workshop on Standardization of Analytical Methodology for Feeds; Ottawa (ON, Canada): International Development Research Center; p. 49–60.

[CIT0038] Weber E. K., Stalder K. J., and Patience J. F.. 2015 Wean-to-finish feeder space availability effects on nursery and finishing pig performance and total tract digestibility in a commercial setting when feeding dried distillers grains with solubles. J. Anim. Sci. 93:1905–1915. doi:10.2527/jas.2014-8136 26020213

[CIT0039] Wenk C 2001 The role of dietary fibre in the digestive physiology of the pig. Anim. Feed Sci. Technol. 90:21–23. doi:10.1016/S0377-8401(01)00194-8

[CIT0040] Williams B. A., Grant L. J., Gidley M. J., and Mikkelsen D.. 2017 Gut fermentation of dietary fibres: physico-chemistry of plant cell walls and implications for health. Int. J. Mol. Sci. 18(2203):1–25. doi:10.3390/ijms18102203 PMC5666883 29053599

[CIT0041] Wu F., Johnston L. J., Urriola P. E., Hilbrands A. M., and Shurson G. C.. 2016 Effects of feeding diets containing distillers’ dried grains with solubles and wheat middlings with equal predicted dietary net energy on growth performance and carcass composition of growing-finishing pigs. J. Anim. Sci. 2016 94:144–154. doi:10.2527/jas2015-9592 26812321

